# Parents’ perceptions of reasons for excess weight loss in obese children: a peer researcher approach

**DOI:** 10.1186/s40900-017-0072-0

**Published:** 2017-11-01

**Authors:** Fiona Gillison, Geraldine Cooney, Valerie Woolhouse, Angie Davies, Fiona Dickens, Penny Marno

**Affiliations:** 1 Department for Health, University of Bath, Bath, UK; 2 Swindon Borough Council, Swindon, UK; 3 Public contributor, Swindon, UK

**Keywords:** Childhood obesity, National Child Monitoring Programme, Parent engagement, Participatory research, Peer-interviewer

## Abstract

**Background:**

This study reports on the process of conducting participatory research by training peer researchers to conduct interviews and analyse data collected with parents of overweight children. The methodology was chosen as a means of (a) encouraging participation among a hard-to-engage group (i.e., parents of overweight children), and (b) generating novel insights and challenging academic/health professional assumptions through the involvement of parents in the interpretation of findings.

**Methods:**

Four parents (all female) were recruited as peer researchers and trained in research processes, ethics, and interview skills over three half-day workshops. The intended interviewees were parents of children identified as obese through the National Child Measurement Programme (NCMP) at the start of primary school (age 4–5) but who had lost their excess weight by age 10–11; little is currently known about how this excess weight loss is achieved. Interviews were conducted by peer researchers, transcribed verbatim and analysed thematically by both peer- and university-based investigators.

**Results:**

The peer researchers felt confident to conduct interviews after three training sessions. Recruitment of interviewees was challenging, resulting in only four volunteers (all mothers) over a 5-month period; thus peer researchers were only able to conduct one interview each. All interviews were considered good quality in comparison to those conducted by Masters-level research assistants. The process of co-analysis resulted in a change in emphasis from that initially generated by the university research team; the role of health professionals in weight management was de-emphasised, and the importance of ‘not singling out’ overweight children accentuated. Given the limited number of interviews, the results of the study are only provisional but resulted in three themes: Whole Family Action, Support (and lack of support), and Protecting Childhood.

**Conclusions:**

Training peer researchers to conduct and analyse interviews was feasible within a short period of training. Peer researchers found the experience interesting, informative and worthwhile. Two of the four volunteered to be involved in a related study 12 months later. The different perspective brought through co-analysis suggests that this approach to conducting participatory research may be a useful means of working with the public to generate new ideas to tackle intransigent issues.

**Electronic supplementary material:**

The online version of this article (10.1186/s40900-017-0072-0) contains supplementary material, which is available to authorized users.

## Plain English summary

Children in England are weighed when they start and finish primary school (aged 4–5 and 10–11 years respectively) as part of the National Child Measurement Programme (NCMP). The number of overweight and obese children increases with age, although some children lose their excess weight. Little is known about how children who are identified as obese when they start school become a healthy weight by the time they leave. The academic aim of this study was to interview parents of children who manage to do this. To encourage greater participation from interviewees and give parents more of a voice in how we investigated and interpreted this topic, we trained four peer researchers to conduct interviews and take part in analysis. The focus of this paper is to report on this method of research. The peer researchers were recruited through online advertisements and flyers distributed through local services. They attended three half-day workshops to learn about research, ethics and interviewing skills. Interviewees were invited to take part via letters from their school nurse, adverts on internet fora and flyers sent to local weight management groups. The interviews were recorded and typed up, word for word. University researchers and the peer researchers first read and re-read the transcripts separately, then came together to identify relevant issues and themes.

Challenges in recruitment led to only four interviewees being recruited (all mothers), so although this allowed the method of participatory research to be tried and tested, the results themselves are limited. The peer researchers found the project interesting, they believed that taking part helped them to gain some useful skills and contacts, and agreed that three workshops were enough to give them the confidence to conduct interviews. The interviews themselves were of a good quality. The peer researchers’ input into the analysis of the interview data challenged some of the interpretations made by the academic researchers, and resulted in different key points being fed back to the public health teams providing childhood obesity prevention services. In conclusion, the study demonstrates how research interviews generating publishable-quality data can be conducted by novice peer interviewers following brief training.

## Background

Research exploring families’ experiences of becoming aware of, and addressing childhood obesity within community populations is challenging as participation rates tend to be very low, particularly among parents of overweight children [1–3]. This study reports on the methodology of a participatory research approach adopted in an attempt to improve participation from this hard-to-engage group. Research specifically exploring parents’ responses to feedback that their child is overweight reports many angry responses, with the feedback often rejected for reasons such as feeling judged negatively by health professionals, and concern that weighing and classifying children as overweight is stigmatising and risks triggering a deterioration in self-esteem or the onset of eating disorders [1, 4, 5]. Further, parents feel that their own priorities for their child’s health (e.g., psychological wellbeing) are not always acknowledged by public health teams that emphasise the importance of weight [2]. As such, it may not be surprising that parents’ willingness to participate in research is low. Given these known challenges to engaging parents around the topic of childhood obesity, this study was designed to take a collaborative research approach incorporating both the public health teams who deliver local services, and parents themselves. The rationale for this approach was based on the premise that parents of overweight children would be more likely to volunteer to be interviewed by other parents than they would by health professionals /academics, as other parents would have greater empathy with the challenges of bringing up children and less likely to form unfavourable judgements.

Childhood obesity is a significant issue for public health, increasing children’s risks of current and future ill health, poor wellbeing and social disadvantage [6, 7]. National monitoring data (such as the National Child Measurement Programme (NCMP) in England; [8]) and international studies tracking body weight over time [9] suggest that most children who are obese at a young age continue to be obese in later childhood and on into adulthood. However, the persistence of obesity is not inevitable; between 10 and 20% of overweight children are a normal weight in adulthood [9] and currently between 11 and 13% of children who are obese in their Reception year (aged 4–5) go on to be a normal weight by Year 6 (aged 10–11) [10]. Practices such as the NCMP, which provide parents with feedback when a child is overweight, are intended as a prompt to encourage parents to make lifestyle changes to help children to reach a healthier weight, but as few parents engage with health professionals as a result of the NCMP [1], how these losses in excess weight are brought about – and whether they are purposefully brought about - is largely unknown. The overall aim of the study was to explore parents’ perceptions and accounts of how formerly obese children have attained a normal, healthy weight.

Past work reporting on the potential benefits of well-conducted participatory research include enhancing recruitment capacity, ensuring research is culturally appropriate [11], the ability to develop a sense of connectedness with interviewees through a better match of language use [12], and encouraging interviewees to ‘open up’ more in relation to sensitive or stigmatizing topics [13]. In line with this potential, parents with experience of, or an interest in childhood obesity were involved in the present study as peer researchers with the aim of providing a more acceptable and comfortable experience for interviewees, and providing insight in the extraction and interpretation of themes from a parents’ perspective. As the study was conceived and developed through an ongoing collaborative partnership between a local authority and university research team, the peer researchers could also act as direct advocates for parents’ perspectives (i.e., study findings) to the professionals responsible for acting on the findings in relation to local services.

This paper reports on a two-stage project to i) train peer researchers to conduct research interviews within their own community, and ii) recruit participants for the peer researchers to interview to explore the research question; ‘What reasons do parents ascribe for the reduction of excess weight in formerly obese children?’

## Method

### Design

The study was conceived and designed through collaboration between the university investigators (FG & GC), public collaborator (AD) and local authority public health team (FD & PM) as part of ongoing research activities to develop ways to address childhood obesity prevention locally. The collaborative team devised and agreed the research question, research design, methods of recruitment, and content of the interview schedule. The research was based in a large town in South West England.

### Phase 1: Training of peer researchers

#### Recruitment

Potential peer researchers were recruited over a one-month period through advertisements in local press, online fora (i.e., volunteering websites, parent fora, and sites advertising part-time work) and from those involved in local authority health promotion services. The advert sought parents with an interest in children’s healthy weights, but it was not a requirement that volunteers had an overweight child themselves. Parents and guardians of children of any age were eligible, as were grandparents with frequent contact with their grandchildren.

#### Peer researcher characteristics

Seven parents responded to the adverts (six women, one man), of whom five attended training and four successfully completed the training. Four were recruited from online advertisements, one through a flyer at local authority children’s weight management services, one through an advert at a local volunteer bureau and one through the university’s existing public involvement networks. The parent who did not complete training was unable to attend all sessions, and did not reach a sufficient standard of interviewing skill within the time available. None of the successful peer researchers had previously conducted interviews or drew on any formal training/support beyond that provided through this project, although two had previously worked in client-focussed roles. All were women: one was a parent of a primary school-aged child who had been identified as overweight and was attending a local authority weight management service, one parent had a 24-year-old daughter with no history of overweight, and two were working-age grandparents with some caring responsibility for pre-school children. The peer researchers’ primary reasons for getting involved were stated as having an interest in promoting healthy lifestyles, an interest in getting involved in research. No formal record was made of peer researchers’ own weight.

#### Training

Training was delivered through three two-hour workshops run during February and March 2016. A final workshop was conducted following data collection to analyse the interview transcripts. The training was designed and delivered by a university researcher (FG), a Public Involvement Consultant (GC), and a member of the public (herself a parent) recruited from a university public involvement group (AD). Workshop 1 provided background to the research topic, set out the aims and objectives of the research, and discussion of how to conduct research into potentially sensitive topics. Workshop 2 focussed on developing peer researchers’ understanding of the research process (including ethics and quality), and provided initial interview skill development exercises. In Workshop 3 further interview skills training was provided, the interview schedule discussed and finalised, and mock interview scenarios using the schedule were conducted. The training materials are provided as a supplementary file. A brief knowledge test was conducted at the end of Workshop 3 to test (and emphasise) important ethical and research-quality related issues (Additional file 1). All peer researchers were offered additional support (e.g., how to deal with interviewees raising challenging content), de-briefing and opportunities to practice and receive feedback on their interviewing skills. Parent-interviewers were paid for their time in conducting interviews at INVOLVE rates [14].

#### Evaluation

Ongoing feedback was collected from the peer researchers after each workshop on (i.e., what was clear, what needed further practice/explanation). Feedback on prepared-ness and confidence was also gathered in telephone de-briefing sessions between the Public Involvement Consultant (GC) and peer researchers after each interview.

### Phase 2: Interview study

#### Recruitment

Parents of children who were identified as obese when weighed as part of the NCMP in reception year (age 4–5), and who were then identified as a healthy weight in Year 6 (age 10–11) were eligible to take part. Initial recruitment was facilitated by the local authority public health team (in partnership with the school nurse team), through identifying eligible parents through their historical National Child Measurement Programme (NCMP) data. Invitation letters were sent to all eligible parents in March–April 2016, and a reminder in June–July 2016 (*n* = 16). The invitation letter made it explicit that they would be interviewed by another parent if they agreed to take part; “*If you agree to take part, you will be asked to meet another parent for a one-off conversation (interview) about your experiences*”. The target sample size was 10 parents. As the initial approach generated insufficient numbers, recruitment was extended through online fora (i.e., volunteering websites, parent fora, and sites advertising part-time work), through existing child weight management services, and advertisement to local members of a commercial weight loss organisation.

#### Procedure

Ethical approval was provided by the University of Bath Research Ethics Committee for Health. The recruitment of study participants was managed by the lead author (FG), who received and screened calls from interested volunteers, explained the study, and talked participants through the consent process. Before assigning a peer interviewer we checked that they were not known to the interviewee. Peer researchers then contacted the interviewee direct to set up a time and venue for the interview to take place; a lone working policy was discussed and implemented; peer researchers logged the time and venue of their interview with a member of the research team, and texted them before and after each interview to confirm their safety (see supplementary materials for the flow-chart provided to clarify this process). Peer researchers could choose whether to conduct interviews in participants’ own homes or in nearby quiet venues (e.g., library, café). Before beginning the interview, peer researchers confirmed who they were (i.e., parents themselves, rather than health professionals) and why they were conducting the interviews (i.e., to try to create a more informal setting, and greater understanding of the issues parents may raise), obtained written consent, and reminded participants of their right to withdraw at any time. All interviewees were left with a sheet providing information about local services and facilities following the interview. Interviews were digitally recorded, transcribed verbatim, and anonymised prior to analysis.

#### Interview schedule

The interview schedule was drafted by the university research team and public collaborator based on past work with parents, reference to published literature, and discussion with local public health teams. The draft was refined with the peer researchers during Workshop 3, after piloting the schedule on each other; this resulted in clarification to wording, but no substantive changes to the questions posed. The schedule addressed four key areas;

1. Whether parents remembered being informed that their child was ‘very overweight’ in their Reception year at school (had they agreed, what had they thought or done in response?),
2. What had happened between Reception and Year 6 that they believed could account for their child’s change in weight status,
3. Whether parents had made any conscious changes to the child’s lifestyle over this time,
4. What strategies they used to try and help their child maintain a healthy weight now.

#### Analysis

The interview transcripts were analysed using thematic analysis techniques [15] in a two-step process. The initial step was led by the lead university-based researcher (FG) who read, re-read and coded key meaning units across transcripts. Transcripts were coded manually by directly annotating each interview transcript (e.g., using highlighters to identify meaning units to which codes were ascribed). Codes were then grouped into clusters, and discussed with the second researcher (GC), who had also familiarised herself with all interviews (Table 1). Discussion focussed on refining the clusters to reduce overlap and ambiguity, while ensuring all codes/themes were represented. This process was undertaken ahead of involvement of peer researchers to reduce the data into a manageable number of categories and topics for group discussion with novice researchers.

**Table 1:** Clusters identified following coding of all transcripts by university-based researchers

Cluster	Description
1. Recognition that a child is overweight	• How the parent became aware that the child was overweight • Memory (or not) of receiving an NCMP letter • Discussions ‘looking back’ at beliefs about the child’s weight at an earlier time
2. Parents’ health beliefs	• Parents’ belief as to whether their child is/was overweight • Parents’ beliefs about whether the child’s weight status will change if nothing is done. • Beliefs about the dangers (or not) of being overweight.
3. Parents’ role and responsibility	• Parents’ views on the legitimacy of measurement /professional involvement in weight loss – should we be weighing children and telling parents their children are overweight in the first place? • Parents’ perceptions of their personal responsibility for making sure their child has a healthy weight.
4. Protection from knowledge	• Shielding child (or not) from knowledge that they are overweight • Concerns around risks to the child’s wellbeing from talking about/explicitly addressing weight in the home.
5. Protecting childhood	• Discussions about the rights of a child to have a ‘normal’ childhood, to do what other children do, without being worried about their weight.
6. Child’s role	• The degree to which parents expect the child to be in control of their choices around weight (e.g., what they eat and drink outside the home) • Parents’ views of the child’s competency to control their eating and drinking. • Degree to which parents feel a child’s weight is the child’s own responsibility. • The degree to which parents involve the child in making lifestyle changes (e.g., gets them involved in food preparation etc.)
7. Social support	• Recognition of the importance of social support (within the family, or from groups) • Peer influences on the child and their weight-related activities (positive or negative)
8. Stigma	• Views of fairness/discrimination against people who are overweight in general. • Discussion of whether weight concern stems from trying to force everyone to be a certain size for aesthetics, rather than for health reasons.
9. Helpfulness of professional support	• Parents’ reports of weight management services and whether they have been helpful. • Parents’ reports of commercial organisations and whether they have been helpful.
10. Barriers and enablers	• Suggested tips and tactics. • Changes that the family has made *on purpose* to try to reduce the child’s weight • Changes that the family has made *incidentally* that have had an impact on the child’s weight • Discussions of how having a child who is overweight has impacted family life. • Barriers to making lasting changes.

Peer researchers were sent all interview transcripts to read, and allocated one transcript to read in detail that was not the interview that they had personally conducted. This approach was taken to provide greater structure and direction to the peer researchers, and ensured that there was an expert (or advocate) for each individual interviewee to check the relevance of the final agreed themes to each individual case.

In Stage 2 of the analysis process, the university and peer researchers met together to complete the analysis, with a local authority representative observing (FD). First, the peer researchers were each asked in turn to provide their reflections on the interviews (“*What are the main messages that you think the parents were making that we should feed back to the people designing and running local services*?”). All ‘messages’ identified through the discussion were added to the list of clusters and definitions generated by the initial coding process (i.e., cluster names in Table 1), with each represented on a separate piece of paper. A prioritisation task was used to provide structure to the process of funnelling all possible insights from both the inductive coding process and peer researcher insights into a manageable and hierarchical set of themes. To do this, a nominal starting point was taken (i.e., a single message/cluster name on a piece of paper), against which each other point was then compared to ascertain (a) if it was substantively different or related to the comparator (i.e., within the same theme), (b) if distinct, whether the new idea was more important or less important in answering the research question than the others on the table, and (c) to ensure that each idea was optimally worded or framed to explain the points discussed.

## Results

### Interview training

#### Peer researcher experience

Four of the five people trained went on to conduct interviews; it was explained to the fifth member of the group that they had not met the level needed within the time they had been able to commit. Through brief debriefing interviews, the peer researchers reported that they believed that the training course had improved their skills and confidence in conducting interviews, and given them insights into the purpose and conduct of research studies. They felt that they gained transferable skills, such as interview techniques and an understanding of research processes, as well as deeper knowledge and understanding of a subject area of interest to them. The most useful training methods were reported to be the listening exercises and role play; role play was found to be initially challenging and off-putting, but all recognised its utility in providing practice of their interview skills after they had taken part. The only concern raised during the process was in relation to the fifth trainee (who did not become a peer researcher), who had not attended all training sessions and demonstrated some lack of understanding of the role of interviewer (i.e., listening rather than advising) which affected the otherwise cohesive dynamic of the group. This influence was mitigated by pairing this person with a member of the training team (AD) rather than another peer researcher for the paired-exercises.

All four peer researchers had felt fully prepared to conduct a ‘real’ interview following training, were enthusiastic to get started, and none took up the offer of an additional training session. No issues of concern were raised by the peer researchers either before or after the training sessions, or before or after the interviews (investigated through de-briefing phone calls). However, they were disappointed that they were unable to fully utilise what they had learnt during the process due to the lack of interviewees recruited, but felt that they had benefitted from the process in terms of contributing to important research, gaining transferable skills, and forming new friendships and business relationships.

### Quality of research output

The interview transcripts were first assessed by the university researchers for interview quality. They were scrutinised for; i) evidence of bias (i.e., leading questions) ii) maintenance of a neutral stand point (i.e., refraining from expressing own opinions, correcting participants), and iii) facilitating elaboration from the interviewee on the topics covered in the schedule. No interviews raised cause for concern regarding bias/impartiality, and all generated relevant interviewee-led discussions. As each interviewer was only able to conduct one interview, it was not possible to explore improvement/change over time. While no formal feedback was sought from the interviewees regarding the person conducting the interview, in follow up correspondence (i.e., providing vouchers for taking part), two interviewees spontaneously commented on how much they had enjoyed the interview, and praised the interviewer’s performance.

The second research activity was the co-analysis of the interview transcripts. Qualitative analysis is inevitably a subjective process, in which the aim is not to arrive at a single true reality, but for which a process of triangulation can be conducted to improve the trustworthiness and dependability of the data [16]. The process of co-analysis operated as an intensive approach to triangulation. The codes extracted by the university-based researchers were acknowledged as appropriate to the dataset by the peer researchers, but the peer researchers presented a different understanding of the relations between the codes extracted, and their relative importance within the context of the lives of those interviewed. The initial 10 clusters identified by the university researchers were supplemented by two factors that the peer researchers did not consider sufficiently salient; 1) lack of long-term support from health professionals (e.g., unhelpful GP, wrong type of support provided by children’s weight management services, long-term support only available from commercial programmes), and 2) the emphasis of ‘not singling a child out’ by responses to weight-concern, which parents believed would feel to the child as if they were being punished.

Following the incorporation of these considerations with the other 10 clusters, the prioritisation process resulted in three overarching themes believed to highlight the main common messages coming from parents interviewed that should be raised to commissioners and those running the NCMP (see next section). Several elements that were identified as interesting/novel by the university-based team were not considered of primary importance by the peer researchers; these included parents’ health beliefs, acceptance that a child was overweight, and parents’ sense of responsibility for helping the child to reach a healthy weight.

### Interview outcomes

After five months of concerted recruitment attempts, only four interviews were completed; two were recruited through letters from the public health team (all eligible parents were sent invitations on two occasions), one from an advertisement online, and the fourth through adverts at a commercial weight loss provider. All participants were women, lived with a male partner, and had children of ages ranging from primary school age to adult. All had at least one child aged 10–11 (the target in relation to receiving a recent NCMP feedback letter) but their comments could relate to their other children too. Three interviewees had attempted to lose weight themselves using a commercial weight loss service over the period during which their child had lost their excess weight, and two had used local authority children’s weight management services at some stage. Two interviews were conducted in participants’ homes, and two in local cafés, and lasted from 21 to 29 min.

Given the small number of interviews, the thematic analysis is considered preliminary, providing ideas for further investigation rather than a set of results to stand alone. The three themes identified were: Whole Family Action, Support (sources and importance of), and Protecting Childhood.

### Theme 1: Whole family action

All of the parents interviewed believed that there had been sustained changes to their child’s diet and/or physical activity level over the course of primary school. While most of these had been made consciously by the parent, the changes had been instigated for the whole family, not just for the overweight child. Furthermore, parents mostly reported the ‘trigger’ for these changes being the parents’ decision to change their own diet or activity levels, not changes instigated for the sake of the overweight child. As such, the impact on the child’s weight was seen as a ‘spill over’ effect, for example through changes to the parent’s shopping and cooking practices.

*P1: And also I went to Slimming World and started doing healthy living and stuff, as he’s not very fussy, so he was just eating what we were eating….. Yeah, yeah we changed our eating habits, like our teas and everything and everything was made from scratch rather than being bought processed and that.*

*P1: We got dogs! So we were going on dog walks. But apart from that…So we did… He didn’t come on every walk with me, but he was doing sort of one 40 minute walk a day? Which he wasn’t doing before.*

*P2: You know, it’s that, that idea from the very beginning, you make sure that they’re all included, you all eat together.*

This decision from a parent to make changes for their own sake came across as a necessary condition for any lasting change to a child’s lifestyle to happen, even when previous effort had been made on behalf of the child. For example, two parents (and their overweight children) had attended children’s weight management services at some previous point in time, but neither parent believed this had had a long-term impact on the child’s health behaviours or weight; they believed that the loss of excess weight was more associated to separate, parent-instigated decisions taken at a different point in time.

*P2: All they did was they gave them the tools to measure the foods, they knew what to eat. And I don’t – and I told them about the exercise, but I don’t think they really had the positive mental attitudes.*

*P4: So we went for that for I think it was like a six-week thing. ….. But erm, it was only a six-week thing. It was only that one. We only did that one. And then obviously we realised she’s putting more and more weight on, so we just decided after Christmas we was gonna’ try Slimming World!*

*P4: Well it [MEND] was just telling us what we should be feeding her and it’s just the healthy eating, yeah.*

Parents’ interpretation of the process of change was that learning that a child was overweight was not seen as sufficient to trigger sustainable changes on its own. Attending child weight management courses (for example) may well have provided education about cooking, diet and exercise at those sessions was later drawn on, whether consciously or not, but parents downplayed the impact of such experiences on the longer term or sustainable changes to the family’s lifestyle that they felt had helped their child to reach a healthy weight.

Parents’ assertion that their child’s weight status was not a trigger for change was supported at least in part by parents’ comments that despite recognising that their child was overweight during early childhood, they had not necessarily believed that the excess weight would persist and therefore did not feel the necessity to act on it. For example, several parents reported considering body size as largely a family trait (“*Cause he’s very much like his dad in a lot of ways, you know, looks and everything. I just thought, ‘he will lose it [weight]…’ in my mind.” (P1))*. Nonetheless, two parents who had not been concerned at the time stated that on reflection they now believed that if they had not made the changes they had, their child would probably have not lost weight - and perhaps even gained weight. Only one parent firmly believed their child had grown out of their excess weight ‘naturally’ (Participant 1), despite describing a number of significant changes to the child’s diet and physical activity that may have conceivably contributed to this.

### Theme 2: Support

Social support was considered very important for maintaining change for both parents (e.g., getting support through commercial weight loss programmes for their own weight), and children (e.g., in making healthy choices when away from home). The emphasis of social support varied in line with the degree to which parents had involved their children in the rationale for lifestyle changes. That is, some parents had made changes without informing their children (“*They are completely unaware that they are that they’re actually following a heal[thy lifestyle]- that- I think, with boys as well, do not tell them they’re following a healthy lifestyle, because they will go the other direction! You – you do… I cheat!*”), whereas others had taken them along to commercial weight loss programmes and had very high expectations of them in making healthy choices (“*She would make her own choice on what she had [from the school canteen]. And you can guarantee it would probably be something without chips. And she would always go for a fruit or a yogurt option”).*

Parents were generally critical of the support given to them by health professionals, and many were unsure where they could go to get support, or what was available.

*P4: Maybe I’d like a support thing for children. Yeah, John’s age. You know, there isn’t a lot about like that…. ‘Cause it does… it can wreck you down, can’t it?*

While the two families who had attended the local weight management scheme (MEND; one was referred by the NCMP, one self-referred) appreciated the opportunities this provided for their children to spend time with others who were also overweight, there was a feeling that the support was too short term, and not focussed on what was important to parents.

*P2: Erm, but we really, we really embraced it [MEND]. It was good to see that the children were helping each other, and I think what it was is that they didn’t feel that they were on their own. ….. And I think, it wasn’t so much the actual MEND program, it was the actual mental attitude. And it was that they had to feel that they weren’t different. That they were even. And I think it is positive mental attitude [that really makes a difference], visualisation, all things like this that they can actually see. All they did [at MEND] was they gave them the tools to measure the foods, they knew what to eat. And I don’t – and I told them about the exercise, but I don’t think they really had the positive mental attitudes. ….That element was missed.*

In contrast, the time-unlimited support from commercial programmes was praised, particularly referencing positive recognition from group leaders and other people trying to lose weight.

Parents also identified peer activities as a threat to weight management, as they believed growing older was associated with children spending time in ways that were not active, and/or being more exposed to tempting high fat or sugar foods;

*P2: That is their youth club - standing outside the chip shop*

*P4: I think the difficulties she has is, she goes out with her friends, they go to a thing called ‘Shake Away’. Yeah, it’s milkshakes made with all, any different chocolate bar you want, and have that made into a milkshake. So obviously that’s high in sugar, but, I suppose it’s hard for her when she goes out with her friends because they all… they all do it and she doesn’t want to be left out.*

Of greater concern, half of parents reported that their child had been bullied because of their weight, or that bullying influenced their child’s weight, and felt that schools could do more to address this. For example, one parent called for schools to deliver education to pupils to reduce weight-related name calling.

### Theme 3: Protecting childhood

All parents expressed a wish to protect children from the stigma or poor self-esteem that could result from feeling ‘singled out’ because of their weight. They felt that all children should be entitled to a worry-free childhood without having to be too concerned with what they eat, and enjoying the same treats as other, normal weight children.

*P2: And that’s one thing that I would pick up on, if anyone ever said anything to a child like that, and making them separate from the group …. and, and labelling them, that’s the worst thing.*

*P3: She’s still 11, she’s got to have the treats for 11-year-olds, so I don’t sit there and say she can’t have chocolate and you can’t have ice cream, and you can’t have that.*

Some parents drew on their own negative experience of being overweight as a child in their decision to try and prevent this from happening to their own children;

*P3: And that’s why I would never ever let her get weighed because I don’t want her coming home being hung up on it, and going the opposite way and not eating. Or throwing up and then you’ve got a bigger situation on your hands…I don’t want her to be hung up, [feeling] “I’m on a diet”. Because I’ve been hung up on my weight for so many years, and it does get soul destroying. Anyone with a weight problem feels the same. I just want her to make good choices.*

Similarly, a parent with an older (now adult) child who had been very unhappy while trying to unsuccessfully lose weight during childhood had tried to protect her younger children by ‘tricking’ them to eat more healthily without their knowledge:

*P2: I… add little cheats, I put…. er, we get a coke bottle, and then I put diet coke in them, and they don’t know that they’re having it. Basically, I lie to my children, it’s awful (laughs)*

Thus, much of parents’ motivation to make changes as a whole family stemmed from wishing to protect their child from potential threats to their wellbeing from concern about the need to manage their weight. While this at first appeared somewhat at odds with the two examples of parents who had taken children along with them to commercial weight loss programmes (thus, unavoidably being aware of their excess weight, and the need to do something to reduce it), this was not feared in the same way as it was considered a ‘normal’ family activity; they were attending with their parent rather than singling them out. In fact, most of the children discussed in the interviews had been aware that they were overweight, either as the parent had told them or they had been teased about it at school, but parents all reported that they had down-played this at home. Changes that parents made to the family’s lifestyle were largely presented as a generic need for all the family to get fitter and to eat better, and not as something specifically directed towards weight loss, or one specific child.

*P2: Keep it positive! Don’t tell them ‘you’re fat’, don’t put any of those labels in. ‘You’re cuddly’ – I said to Sarah you know, ‘you’re just cuddly’ and, you know, let’s get fit so that we can… do activities, not ‘cause you can fit into a dress size’…*

## Discussion

This participatory research project demonstrated that it was possible to train novice interviewers within three, two-three hour workshop sessions to a point where they felt competent to conduct interviews with their peers. Four interviews were conducted that met the quality requirements of data collected without evidence of undue bias or influence, and providing sufficient depth for analysis. It would be expected that the quality and depth of interviews would have improved further had the peer researchers had a chance to conduct further interviews. It was not possible within this research design to assess whether this approach resulted in the benefits reported by other studies [11–13] in relation to the openness and comfort of participants, but both interviewers and interviewees reported enjoying the experience. Further, the peer researchers believed they had personally benefited from enhanced skills and networks as a result of taking part and two of the four accepted the invitation to take part in further interviews in a related project 12 months after the initial interviews.

From the university-based researchers’ perspective, the process of collaborative analysis was positive, insightful, and constructively challenged their initial assumptions. We considered two key factors in evaluating the efficacy of this method; first, maintaining scientific rigour, and second, generating insightful, high-quality results. In relation to the first consideration, we were concerned to ensure that any method of analysis chosen would allow the involvement of peer researchers without either placing undue burden on them or sacrificing the systematic and unbiased consideration of the raw data. The two-stage approach allowed us to do this efficiently; through Stage 1 we ensured that standard coding procedures were used to extract the full range of meaning units from all transcripts by researchers trained in qualitative methods, and in Stage 2, peer researchers contributed to the process of combining and prioritising these into themes. The requirement on the peer researcher time was thus limited to 3–4 hours (attendance at a workshop, plus prior reading).

The second consideration of generating insightful results is hard to quantify. However, considerable differences were seen in the focus and tone of the initial themes generated in Stage 1, and the final themes agreed following Stage 2 which suggest that a meaningful process of re-framing the raw data to a more parent-focused perspective took place. The peer researchers prompted greater consideration of where parents had identified the support offered as lacking or unhelpful relative to commercial weight loss providers, giving much more attention to parents’ perceptions of how unhelpful weight-loss services and advice had been and flagging up characteristics of commercial programmes that they felt service providers could learn from. Similarly, the peer researchers clarified how the sometimes contradictory statements parents made about protecting their child versus engaging them in weight management activities could be reconciled by thinking of these activities in relation to ‘not singling a child out’. These discussions challenged perspectives of what activities within families are currently considered normal (e.g., attending commercial weight loss programmes) versus setting children apart (e.g., lack of provision of snacks). In contrast, some themes highlighted by the university-based researches, such as parental knowledge about healthy lifestyle and their awareness of a child’s weight status, were considered less important by the peer researchers in terms of finding a route towards better support for families.

In reflecting on what aspects of the method used were facilitative of this process, the key factors were considered to be the creation of a climate in which peer researchers felt able to make suggestions and challenge ideas freely, and the provision of sufficient structure to prevent the task from appearing overwhelming. Presenting all ideas on pieces of paper was considered useful in giving university- and parent-led ideas equal footing, and encouraging peer researchers not to feel that any ideas generated by the university-based research team were fixed and needed to be retained (they could be written, rewritten and discarded at will). Similarly, by asking the peer researchers to provide their impressions of the key themes to come from the interviews in their own words before reading the university-researchers’ cluster labels, the discussion was made more accessible by initiating debate in lay- rather than academic terms. The specific prioritisation approach used to generate themes from the initial dataset represented on the paper notes was not essential to the process, but provided a useful structure to create a clear, comprehensive and flexible focus for group discussion.

### Interpretations of the findings

The analysis of the interviews themselves generated insight into some of the beliefs that parents have about why children lose excess weight during their primary school years. One finding highlighted by the peer researchers was the assertion from those interviewed that the trigger to lasting positive weight change had not come from an awareness of the child’s weight status, or the influence of advice from health professionals. This was the case even among families who had engaged with children’s weight management services. Instead, parents attributed lasting change to decisions they had made to tackle their own weight that had resulted in changes to the diet and activity habits of the whole family. It is possible of course that these experiences (e.g., the advice and education provided) may have influenced the changes parents made at a later point in time without them being conscious of this link, but even if so, parents’ strong belief that this was not the case suggests that these contacts were not having a direct positive impact as intended. The three themes converged to suggest that parents found it inappropriate to focus on influencing a single child’s weight or health behaviours, and that awareness that a child was overweight was insufficient to trigger whole-family changes. In part, this latter point may reflect parents’ lack of belief at the time of the first NCMP measurement (at age 4–5) that being very overweight was a significant concern [3, 5]. This was referred to both in relation to the belief that their child would simply ‘grow out of it’ in due course, and lack of belief of the severity or inevitability of potential long-term threats to health.

Parents’ preference for a family-based approach to weight management, including sustainable changes to physical activity and eating patterns and the involvement of a child’s wider family, is consistent with the model proposed by public health teams [17]. As such, there does not seem to be a mismatch between what children’s weight management services are recommending that parents do, and what parents find appropriate and acceptable. Instead, the mismatch is between beliefs about when action is called for and how it should be framed. Parents in our study did not consider a child being obese in young childhood to be sufficient reason for the child or family to instigate change. Conversely, a parent wishing to lose weight did appear to be a sufficient trigger for change in family lifestyles. It is perhaps understandable that parents prefer to attribute the decision to make lifestyle changes to the health or weight of adults, rather than children, given the pervasive concern to protect their children from harm (e.g., contributing to a child’s undue weight concern, poor wellbeing and self-esteem, consistent with past work; [2, 18, 19]). This may particularly be the case as children get older, and parents therefore need to engage their child's support for such changes to be accepted and abided by.

The impact of parental awareness of a child’s weight status or health behaviours shows some contradictory associations in past work. For example, in support of the importance of parental awareness, parents’ responses to the English NCMP indicate that a good proportion of parents report an intention to make changes, or self-report having made changes soon after feedback that their child is overweight [19]. In contrast, data from the prospective Gateshead Millennium Study (conducted in northern England at a similar point in time) showed no association between parents’ awareness of overweight in seven-year-olds and the child's BMI at age 15 [20]. Our findings show some evidence that could be consistent with both of these effects; two families had attended a local child weight management programme (i.e., short-term changes and intentions following engagement with health professionals), but found that doing so was not sufficient to bring about lasting weight loss (i.e., lack of translation to long-term weight or behaviour change). Thus, raising parental awareness of overweight may bring about a parental readiness for action that has the potential for positive outcomes for the child, but if this potential is not realised through directing parents to child- rather than family-focused interventions, this impetus is lost. Our findings suggest parents are unlikely to consistently enforce any change that effectively singles a child out from their peers (thus reducing the efficacy of child-only approaches), and a greater impetus is needed for a parent to decide on family-wide lifestyle change than a single child’s weight.

### Strengths and limitations

Facilitating public involvement in data analysis (rather than solely in data collection) was a strength of the study, by allowing parents’ perspectives to take priority in the interpretation of the interviews. This was not least as some of the peer researchers had personal experience of a child or family member being overweight so could reflect on the views of others through the lens of their own lived experience. The process of integrating the two perspectives therefore allowed us to arrive at a more parent-focussed set of findings than would otherwise have been the case. Although we recruited other parents to conduct the interviews (and publicised this in recruitment materials), this was not a fully participatory research project; in line with a critique of other community-based participatory research [13] the peer researchers were engaged only after the public health and academic collaborators had set the research question and approach. A better approach may have been to recruit peer-researchers or an advisory group of parents (e.g., through parenting groups online or based at a school) earlier in the process to enable them to be co-creators of the research question and study design, which may have been more relevant and interesting to the parents themselves. While such a ‘bottom up’ approach may be desirable, it is challenging to facilitate this when funding for public involvement is largely awarded after applications for a specific research project, involving such decisions, have already been made. Members of the public may not always be keen to be involved in all stages of research. For example, only one peer researcher was interested in drafting this paper, and this is because she has a professional interest in writing and editing; others were interested only in taking part in the more active aspects of the research.

There are also limitations of the participatory approach taken: It may be more challenging for peer researchers to remain objective/ avoid bias where the outcomes of research have personal relevance (e.g., if the findings could influence services in their area), although steps were taken in the present research design to ensure that interviewers did not know their interviewees, and that the systematic coding of all interviews was conducted and incorporated in the analysis. In addition, we factored in time at the start of the first session to allow peer researchers an opportunity to voice their opinions about the topic, and hear each other, before discussing why it may be important that these opinions are put to one side during the research process. Although the interviews showed a good level of discussion and absence of leading questions, they were relatively short (21 to 29 min), and it is likely that more experienced interviewers would have been able to facilitate more in-depth discussions. However, this limitation is in part due to a lack of experience, rather than limitation of the approach itself.

A limitation of the potential academic contribution of the present study was the small number of interviews conducted. Such challenges in recruitment are not unusual, and indeed past work in recruiting parents to feedback on the NCMP process shows response rates of 7–10% [1, 2], lower in parents of overweight or obese children than those of a healthy weight [1, 3]. A range of different recruitment approaches were used, from personal letters to those known to be eligible to advertising online and seeking the assistance of a commercial weight loss programme. This was a limitation not only in the degree to which the findings can be taken to reflect a broad range of parents, but also as it meant the peer researchers did not have the opportunity to conduct more than one interview each and thus improve their skills. As a result of the limited sample pool, this study does not provide a broad overview of parents’ experiences, but a snapshot of experiences of a small number of parents (mothers) in one local area. Expanding the participant pool to include fathers, and a greater range and diverse spread of parents, would improve the breadth and dependability of the findings.

Use of snowballing as an additional means of recruitment was considered, however it raises ethical challenges if asking parents to approach others that they judge have had a child who was previously overweight without evidence that this was the case. On reflection, we believe the poor recruitment rate negated our assumption that recruitment would be easier as we believed we were targeting parents with a ‘good news’ or success story to tell (i.e., their child had regained a healthy weight). This was not how the parents we spoke to saw their experience; the initial judgement of ‘very overweight’ had not been particularly concerning to them, and the fact that their child reached a healthy weight at a later age was not considered an achievement.

A final limitation was the lack of formal evaluation of the quality of the data generated through this participatory research process. We anticipated the possibility that not all volunteer investigators would reach a good standard of interviewing; the recruitment materials were clear that training would not automatically lead to a role as an interviewer, and a brief quiz that participants were required to pass was used at the end of the training session to emphasise key points of ethical practice. However, a more systematic approach of evaluating interview quality, including (for example) gaining feedback from interviewees could have provided more evidence of the sufficiency of the training.

## Conclusions

This study illustrates how members of the public can be trained to conduct research with a modest investment of time, and how their input in the analysis of findings can be facilitated. The process generated good quality interview data, and the process of analysis proved manageable to the investigator team without posing too great a demand on their time. Despite frustration that there were not more interviews to be conducted that would allow them to fully develop their skills, the peer researchers remained positive about the project, with two attending a workshop held with public health professionals across three local areas to provide the parents’ perspective and committing to conduct interviews for future projects. The involvement of peer researchers in the interpretation of the findings facilitated insight into the gap between professionals’ (including academics) and parents’ assumptions about the most important considerations surrounding managing children’s healthy weight.

## Additional file

Additional file 1: Peer Interviewer Training Pack. (PDF 377 kb)
