# Supplementary material for: Parents’ perceptions of reasons for excess weight loss in obese children: a peer researcher approach
Source: Res Involv Engagem. 2017 Nov 1;3:22. doi: 10.1186/s40900-017-0072-0 (PMC5664432; doi:10.1186/s40900-017-0072-0)
Supplement: Additional file 1: — Peer Interviewer Training Pack. (PDF 377 kb) [file 40900_2017_72_MOESM1_ESM.pdf]

Peer Interviewer Training Pack

This pack comprises materials and reflections on using them obtained through running the 'Parents Interviewing Parents' project in January – October 2016.

Fiona Gillison & Geraldine Cooney

Contact: Fiona Gillison: f.b.gillison@bath.ac.uk

Contents:

Background	page 3
Notes on preparation and recruitment	page 3
Evaluation	page 4
Workshop plans	page 6
Appendices:	page 12

Contact: Fiona Gillison: f.b.gillison@bath.ac.uk

Background to the *Parents Interviewing Parents* project

This project was funded by the University of Bath Public Engagement Seed Fund. The primary aim was to pilot a peer interviewer training course and materials to equip members of the public to conduct interviews of research quality. The associated secondary aim was to explore whether using peer-interviewers helped us to gain access to a hard to reach population and led to more open, honest and comfortable discussions within interviews.

The following account was provided to stakeholders and potential project partners, which sets the scene for this training programme:

Childhood obesity is a significant issue for public health, increasing children's risks of current and future ill health, poor wellbeing and social disadvantage. National monitoring data shows that while most children who are obese at age five are also obese at age 11, a minority (around 12%) show a significant *decrease* in excess weight, jumping from the classification of obese at age 5, to a healthy weight by age 11. As few parents engage with health professionals in managing childhood obesity, how these improvements are brought about is largely unknown. Parents' reluctance to engage with health professionals around childhood weight issues seriously restricts our ability to gain access to this population and conduct useful research in this area. Our own (and others') past research suggests that parents of overweight and obese children find receiving feedback that their child is overweight upsetting; they feel judged by health professionals, are concerned about stigmatising their child, and feel their own priorities for their child's health (e.g., prioritising psychological wellbeing) is not always acknowledged by public health teams (Gillison et al., 2014).

This project will involve training members of the communities we wish to engage to carry out interviews, acting as peer researchers. We will identify 4-6 volunteers for the research role through advertising on internet sites and from families currently engaged with public health/school nurse services, and provide training over 3 workshops in research conduct, ethics and interview skills. Trained parent-researchers will then conduct 10 interviews with *study participants* (i.e., parents whose children have lost significant amounts of excess weight) to explore their views on the factors within the family and wider environment that may have influenced this change.

Preparation and Recruitment

Establishing an advisory panel

We recruited an advisory group comprising the two project workers, a public collaborator, an independent academic advisor (experienced in participatory research), and two local authority stakeholders. The Council partners facilitated our use of accessible local training venues, and maximised the chances of the research findings being adopted. The panel met in person at the start of the project, were consulted via email for queries and sent updates during the project. They met again at the end of the project to discuss results and dissemination.

Ethical approval

Our application for ethical approval included a description of the processes we went through in recruiting, training and supporting our peer interviewers as well as the safeguards in place for interviewees. While ethical approval is not necessary for training courses, we considered it important that the steps we put in place to protect and support both interviewers and research participants were approved and scrutinised by the committee.

Recruitment:

Activity	Person responsible	Response (conversion rate)*
Flyers distributed by service providers (project collaborators) to known relevant parents	Service providers (council employees)	1 (1)
Advert on Gumtree (ads can be free, but £19 paid to get ours prioritised)	UoB team	8 (3)
Advert on Mumsnet (£30 for 1 month)	UoB team	0
Position advertised by the local volunteers centre	UoB team	5 (0)**
Existing public engagement partners	Bath Research and Development Participate Network	1 (1)

* Main figures denote no. of contacts, figures in brackets indicate the number who attended from these sources. ** This may be useful if given more of a lead in time - we continued to get enquires after the closing date.

Equipment:

- Powerpoint projector (and laptop/computer)
- Flip chart paper (workshop 1)
- Participant folders (added to each week)
- Travel expense forms
- Petty cash (for people preferring repayment up front)
- Attendance sheet
- Refreshments

Evaluation and Dissemination plans

We planned to examine whether this relatively short (and therefore potentially affordable) Peer Researcher training model provides a feasible, acceptable and effective means of enabling the public to become meaningful partners in the research process. Indicators of success were specified in relation to peer researcher (public) experience, research quality and wider public impact. The advisory group offered ongoing support and oversaw the evaluation plans and processes.

Contact: Fiona Gillison: f.b.gillison@bath.ac.uk

PEER RESEARCHER EXPERIENCE:

Information	Source / Evidence
Perceptions of the pace and volume of information presented across workshops	Informal feedback, and brief questionnaire responses following each session
Perceptions of the research process and the peer researcher role e.g. support provided, opportunity to influence or lead	Post-course debrief (telephone conversations)
Challenges, benefits and risks/costs	Post-course evaluation sheet and verbal debrief (telephone conversations)

RESEARCH QUALITY:

Information	Source / Evidence
Consistency of interview schedule implementation	Evaluation of transcripts
Incorporation of findings as pilot data for future collaborative research grant funding bids	Inclusion of data within submitted bids – and success of these bids
Publication of research findings	Publication (yes/no)

PUBLIC IMPACT:

Information	Source / Evidence
Perceived relevance to public health partners	Evidence of by dissemination within partner's own organisation/networks<br><br>Changes to practice
Subsequent public engagement activity around this issue e.g. dissemination of findings by participant researchers, invitations to speak to other groups	PI to set up monitoring system to record this

Dissemination

This project and its findings will be publicised widely through;

- BRD's Participate Network (105 members of the public interested in getting involved in research)
- provision of a case study report for dissemination through the Public Engagement Unit
- academic publication
- dissemination through public health networks.

Contact: Fiona Gillison: f.b.gillison@bath.ac.uk

Workshop 1

	Time	Notes
**1. Introductions**<br>- Names and motivation for getting involved	10 mins	- Quite quick as group did not know each other.
2. **Introduction to the project**<br>- Background to the topic area,<br>- what we're aiming to do and why<br>- what the findings will be used for	20 mins	- Accompanied by slides<br>- Comprehensive information sheet had already been sent to all, so few questions
**3. Introduction to research**<br>- What is research - introduction to principles and process<br>- Qualitative research – a conversation with purpose	10 mins	- Accompanied by slides<br>- Not an in-depth discussion, but more to demystify and put trainees' minds at rest
**4. What makes a good qualitative interviewer?**<br>- characteristics of a good interviewer<br>- role of interviewer vs educator<br>- asking open questions	30 mins	- Group activity, discussed and recorded on flip charts in pairs<br>- Compared with slides afterwards
**5. Initial activities**<br>a. The 2 minute challenge<br>b. Reflective listening	30 mins	- See session slides for details (and handout Appendix 2)
**6. Feedback**<br>- What was clear, what was 'muddy', what would you like more of?	10 mins	- Trainees didn't have much to report at this point
	1 hr 50	

Reflections:

This was a relaxed session that took less time than we had planned for, but this was in part as our trainees were not very vocal. They had much less to say than the BRD Healthy Living panel who we had previously worked with on this topic (2 of whom joined us in workshop 2). We would not have moved onto reflective listening if there had been more discussion during the session (we had not planned to do so at this point). Overall it was a very positive atmosphere, splitting trainees into pairs worked well as by the end of the session everyone had got to know at least one person quite well, creating a positive group dynamic and bonding that facilitated activities in later sessions. All our participants were willing to do the exercises – and we did them ourselves alongside to facilitate an informal and relaxed atmosphere, reducing barriers between leaders and course attendees. This first session was important for establishing trust, especially as childhood obesity is a sensitive issue.

Workshop 2

			Notes
**1. Recap /questions**	15 mins	-	Accompanied by slides
**2. Research process**<br>- Quality<br>- Ethics and conduct	15 mins	-	Accompanied by slides
**3. Experience of being a researcher for the first time**<br>- Sensitive interviewing<br>- Closeness to the subject<br>- Handling distress/anger<br>- Being prepared	25 mins	-	Presentation by a member of the BRD Participate Network (i.e,. public collaborator)
**4. Interviewing question**<br>- Open questions – provide a handout of open questions and prompts and probing questions<br>- Listening and responding –go through some examples of neutral/empathetic responses	20 mins	-<br>-	Trainees given a handout/crib sheet of open questions (see Appendix 3) to use<br>Recap of the use of reflections and reflective listening
**5. Skills practice**<br>- Use the crib sheets to practice on each other	30 mins	-<br>-	Split into groups of 3 to practise getting people talking using open questions and reflections; see session slides for detail.<br>Trainers rotated to listen in and provide feedback.
**7. Feedback from parents**	10 mins	-	This was much shorter, due to overrunning of practice
	1 hr 55		

To take home:

- Questions crib-sheet
- Interview script for project interviews (to look at ahead of next week)

Reflections:

There was a lot to fit into this session, so more time may have been useful. Some trainees found the simulated interviews/practice with open questions difficult to do and/or felt uncomfortable taking on the role of interviewee at this stage. We had proposed that they talk around a real topic - so the interviewer gets to deal with genuine responses, however some were more comfortable adopting a fictional story. The participants would have preferred a set of clear ‘interview questions’, but our aim was to get them talking, asking open questions in their own words in response to their interviewee, and getting people to expand on what they were saying rather than moving on. If practical, we would recommend making this session longer (2.5 to 3 hours) to fully explore the use of reflective listening, and to allow discussion/ between participants on their skills practice.

Our public collaborator was present at this workshop and presented a personal account of being a novice interviewer. Participants said they found this extremely useful. The involvement of our public collaborator also helped diminish barriers between ‘experts’ and ‘novices’ and create an atmosphere of mutual learning and respect. An additional course member joined in week 2 (having got the day/date wrong). Whilst bearing in mind the sensitive nature of the subject and the fact that some participants had personal experience of obesity, we had perhaps underestimated the importance of bonding and trust that had occurred in Workshop 1. The introduction of a new (and forthright) person caused some difficulties with group dynamics, particularly in the role play exercise. This was dealt with in the session by the trainers, who took steps to mitigate these effects in Workshop 3. In retrospect, workshop attendance from week 1 should be mandatory (this participant was deemed not ready for interviewing after only 2 sessions).

Workshop 3

			Notes
**1. Recap / questions**	5 mins	-	Accompanied by slides
**2. Discussion of the interview script**<br>- How closely should this be matched<br>- Questions, comments and suggested amendments	15 mins	-	Incorporated discussion of which sections may lead to difficult conversations, and how to phrase/discuss in a non-judgemental manner.
**3. Role play the interview with script**	30 mins	-<br>-	Split into pairs, practising different parts of the script.<br>Trainers rotated to listen in and provide feedback.
**4. Dealing with difficult situations**<br>a) someone who hardly talks/ gives one word answers,<br>b) someone who gets angry and starts ranting /makes personal comments	30 mins	-	Trainers played the role of 'difficult interviewees', rotating between groups
**5. Discussion of interview protocol/procedure**<br>- Confirm the required steps<br>- Confirm support we will give	10 mins	-	Accompanied by handout (Appendix 4)
**6. Feedback**<br>- Completion of knowledge quiz	10 mins	-	As per ethical approval, tested understanding of confidentiality
	Total: 2 hours		

To take home:

- Step by step Interview protocol/procedure sheet
- 'Leave behind' sheet of information to provide to interviewees
- Copies of the information provided to participants (including consent forms)
- A personalised Certificate of Interviewing skills/competency signed by trainers

Reflections:
The trainees rose to the challenge of role play, but were more comfortable with us playing the interviewee than role playing with each other. We broke the practice into short snippets – i.e. we challenged them for roughly 5 minutes through playing difficult interviewees, but then allowed a break for discussion (feedback) and rotation between them. We offered trainees a further skills training session the following week, but they chose not to take this option. Had time permitted, we would have liked to have added practice using the digital voice recorders. Ideally, we would make this session longer (as per workshop 2) to provide more flexibility and observed interview practice. A course certificate was given to recognise achievement and commitment, and could be used by participants for future work/training.

Workshop 4

PREPARATION:

PEER RESEARCHERS: Prior to the workshop all peer researchers were sent the transcripts of all of the interviews completed. They were each allocated one transcript to read in detail – which was selected to be a different one from the one they had conducted (i.e., so that they would be familiar with at least two interviews, and had the opportunity to familiarise themselves with all of them if they chose to).

UNIVERSITY RESEARCHERS: In parallel, the lead researcher had coded all interview transcripts as per usual practice in qualitative research, and discussed initial clusters with the second researcher (Appendix 7). This was conducted to try and reduce the data into a manageable number of categories for discussion. The title of each category was then written on a post-it note.

			Notes
**1. Welcome, overview of session**	10 mins	-	Peer researchers were invited to share their experiences of conducting the interviews
**2. Peer researcher feedback**<br>- What are the key messages for public health teams to know?	25 mins	-	Before the researchers shared their framework, the peer researchers were asked to highlight what the key points were from their reading. Any of these that did not overlap with items on the framework were written onto post-it notes for part 3.
**3. Framework presentation and implementation of a prioritisation tool**	40 ins	-	The university researchers presented and explained their framework of 10 clusters. Each was written onto a post-it note, alongside the key points raised by the peer researchers in part 2.
		-	PRIORITISATION TOOL: Starting with one post-it note in the middle of the table, each remaining cluster was compared to it for the group to discuss and judge;<br>(a) whether the incoming cluster was conceptually distinct form the others present,<br>(b) whether the incoming cluster represented a more or less important message than from parents, and<br>(c) if it was correctly framed to explain the points raised. (See Appendix 8)
		-	Cluster names were adjusted and merged where appropriate, and then ranked, until all were allocated.
**41 Conclusions**	15 mins	-	Summary of key feedback points
	Total: 1.5 hours		

POST-SESSION TASKS

Following the session, the university researchers led in compiling the ideas generated during the discussion into coherent themes, and selecting illustrative quotes to support these. The findings were then circulated to all involved for input and approval.

Reflections:
The post-it note system worked well in engaging the peer interviewers in discussion as to what were the most important points, and what the different clusters meant to them (i.e., rather than to the research team). All were engaged and willing to contribute – combining and refining meanings from suggestions made by both university and peer research team members.

Through getting the peer interviewers to provide descriptions in their own words, and debate whether two similar constructs were similar or not, greater clarity and understanding was generated. Some of these discussions challenged the thinking of university researcher and public health professional thinking by providing fresh angles to the interpretations of the data – free of academic . There was also a service provider/commissioner present, which helped to focus the discussions on '*what is it that the parents we interviewed would want this person's team to know*'?

The specific task used (post-it note ranking) was not necessarily important in itself, another approach could have worked equally well in all probability; in fact, the process ended up more of a clarification and compiling of themes, rather than prioritising, but it provided a useful starting point. The main function of the task was to provide a clear, flexible focus for group discussion (i.e., descriptions and cluster names could easily be rewritten, which encouraged suggestions for refining ideas), which it facilitated well. If the session had been too open (i.e., no initial suggestions for clusters had been provided), we believe it would not have been manageable to reduce the data to three key themes (as we did) within a reasonable timeframe. If the university researchers had not conducted initial coding, we would also have lost out on the perspectives of those who had read all transcripts thoroughly – as it was felt too much to require peer researchers to read all before attending. This way, at least one of the peer researchers had read or conducted each interview contributing to the analysis, as well as both university researchers, which allowed the interpretations of the university researchers to be checked and validated/challenged by members of the public. Thus, the process allowed all potential extracts to be raised for discussion, before the final set were agreed with the peer research team.

Appendices

Appendix 1: Trainee information sheet

Appendix 2: Reflections worksheet

Appendix 3: Open questions crib-sheet

Appendix 4: Procedural flow chart

Appendix 5: Post-training quiz

Appendix 6: Post-sessional feedback sheets

Appendix 7: Analysis framework

Appendix 8: Process of ranking and theme generation

Appendix 9: Session 1 slide set

Appendix 10: Session 2 slide set

Appendix 11: Session 3 slide set

Appendix 1: Trainee information sheets

Healthy weight in children: Learning from parents

Information for potential interviewers

Thank you for your interest in our interviewing project. The scheme has been funded by the University of Bath's public engagement team, to see whether we can improve our research by getting the public more involved. This sheet gives you more details about what it would mean to take part.

Why is this study being run?

We often see stories in the news about the large number of children who are overweight, and the problems this may raise for them in terms of their health, wellbeing and how they feel about themselves. The government and local councils are putting a lot of money into finding ways to help more children to keep to a healthy weight. But we have not managed to do this yet, so are always looking for new ways to find out what works. This project is a small-scale study to talk to parents whose children lost weight in primary school, so we can learn about what worked for their family.

Being overweight can start at a very young for some children - about one child in five is overweight at 5 years old. This increases to one child in three by the age of 11. We know that most children who start out life overweight, tend to stay overweight. But a small group of children go against this trend; they are overweight at age 5, but they have reached a healthy weight at age 11. We think these children are a really interesting group – how do they lose their extra weight? This study aims to try and find out what sort of changes may be behind this. The parents we recruit as volunteers to be interviewed will be the parents of children who have lost their 'extra' weight over the primary school years.

What would my role be?

You have answered our advert to be a '*parent interviewer*'. We know that parents may not be comfortable talking to health professionals about what goes on at home that might affect a child's weight, and can feel judged by them. We wondered if it would be easier for them to talk to other parents instead – people who may be from a similar background or area, and who they may feel have a better understanding of what it's like to bring up children. People who won't judge them for being 'less than perfect'.

So we are offering some basic interviewing training to parents, grandparents and carers living in the Swindon area to go out and be our eyes and ears in learning more about this. We will be asking you to talk about what the challenges are for parents of overweight children, and what has worked well for these families to help move their child towards a healthy weight.

What will taking part involve?

To be a *parent interviewer* you must attend three 2-hour training sessions run by us in central Swindon. We will pay your travel expenses to get there, but unfortunately we can't pay your time while you are there.

The sessions will involve training in general research skills (things like ethics, and how we make sure that we have interviews of good quality across all our interviewers), and training in interview skills. At heart, an interview is a conversation with a purpose, so anyone who can happily talk to friends and family about important issues should be able to do an interview. The training will focus on how to ask open questions, how to make sure you come across as neutral (not judging), and some hints and tips of what to do if the person you interview is very quiet, very talkative, or difficult to keep on track.

We will then give you an interview script – that is, a list of questions that we ask you to go through with each person you interview. This way, everybody gets asked similar questions regardless of who does the interview. We will give you some practice with this script in the training sessions before you are sent out to do any 'live' interviews.

After the training, you will be matched to people volunteering to be interviewed, and asked to arrange a time and place to meet that is convenient to both of you. This may be in the other person's home, or it may be in a quiet public place. You will have a choice of whether or not you do interviews in other people's homes, and in how many interviews you take on. Interviews usually last for around one hour, and you will be paid for your travel and interviewing time at our standard rates (approx. £13.18 /hour - or you can choose to take the equivalent amount in vouchers if this would put any of your benefits at risk).

We will need to record the interviews so that we have a record of what was said. Only the person typing up the interviews will listen to the recording; once we have checked the recording we will delete it. If names are mentioned in the interview, we will change them. When we report the findings of our study, we will make sure that neither you nor the person you are interviewing can be identified from what is said.

We have some simple digital recorders that we will demonstrate at the training sessions and you will use to record the interviews. We will ask you to post these back to us after each interview you do so we can download the interviews.

How much responsibility will it be?

We will give you a lot of support during the training and running interviews. We will also be careful about your safety; one of our team will know where you are carrying out each interview, and check in with you before and afterwards, to check everything is OK. We will speak to you after each interview to give you a chance to 'debrief' – that is, talk about what went on,and give you a chance to ask about anything that came up that you weren't sure about.

We will expect you to stick to our rules about confidentiality, which we will tell you about in the training. We will ask you to sign a form to say you will keep any information you learn about the person you interview confidential, and that you will keep the names of the people that you interview confidential.

How many interviews will I be asked to do?

We don't know how many interviews there will be in total, and we don't know how many people will finish the training, so we can't give you exact numbers at this point. We don't expect more

than 15 or so people to volunteer to take part – and it may be less. So even if we end up with very few *parent interviewers* like you, each person will not be asked to do a lot of interviews.

***Taking part in the training does not guarantee that you will be asked to do any interviews*.**

As we have to keep an eye on the quality of our research, we will only ask people who are confident and capable by the end of the training to do the interviews. It may be that everyone we train is successful – after all, you don't have to be perfect to do a good job. But it is important to say up front that we cannot guarantee that you will be asked to interview anyone.

Also, we cannot guarantee that we will be able to find enough volunteers to be interviewed in the time we set aside, or at times when you are free. So the number of interviews you do may also be limited by your availability and the availability of people to interview.

What if I decide I don't want to do any interviews after the training?

That's fine by us. As long as some of the group are happy to do the interviews, we don't expect everyone to take part at the end. Please tell us if you would prefer not to carry or if it is more difficult to fit in to your life than you realised. Interviewing is not for everyone, and you won't know how you feel about it until you have done the training.

But we hope most people will enjoy the training and be keen to take part!

What now?

If all the information above hasn't put you off (!), then all we need to do is check whether or not you can make the training dates. Unfortunately we can't be very flexible on this. However, if you are interested but find you can't commit right now, we will still invite you to our 'RESULTS' event once all the interviews have been recorded, where you will have a second chance to have an input.

Training will take place at…..

.

Contact details:

UNIVERSITY OF BATH

Appendix 2: Reflections worksheet

Reflections and probing worksheet

Have a go at coming up with one ‘probing’ question (i.e., asking more), and one ‘reflective statement’ that you could use to answer each of the following, remembering that:

- a reflection is you repeating what you have heard
- reflections are often paraphrases
- reflections end in a full stop.

Use probing questions to check your assumptions and encourage people to expand and tell you more. Use reflections to show that you understand (show empathy)

---

1. *I’ve got no idea why he lost the weight – nothing changed at home.*

2. *I wasn’t going to make any changes, as that would have made it worse for my daughter.*

3. *I mean, everyone knows that BMI isn’t the right way to tell if a child is overweight or not.*

4. *She was really active and ate a healthy diet, so I didn’t see why I should be worried.*

5. *Even then (when she was 5) I did everything I could to try and cut down on what she ate, but you know how it is, there’s no arguing with kids.*

6. *He just hated sport so what was I gonna do?*

Appendix 3: Open Questions Crib Sheet

OPEN QUESTIONS

These example open questions encourage interviewees to talk and think about their experiences/something that has happened to them

- *Thinking back, what do you remember about…*
- *When did you first notice something….?*
- *What did you do when…?*
- *How did you react to/feel/think …?*
- *How have things been since then…. or How are things now?*

PROMPTS - to encourage more talking, when people ‘dry up’

Prompts help interviewee remember what happened, give examples or more details

- *What happened next…?*
- *What do/did you think about that...?*
- *Do you remember any more about,,,?*
- *Some people have said/think….*

PROBES – for more detail or when something needs clarifying

Probes look for reasons, explore what has been said in more depth

- *You said earlier…?*
- *What effect did that have on you…?*
- *What makes you say that…?*
- *I want to make sure I understood correctly…*

Appendix 4: Procedural flow chart

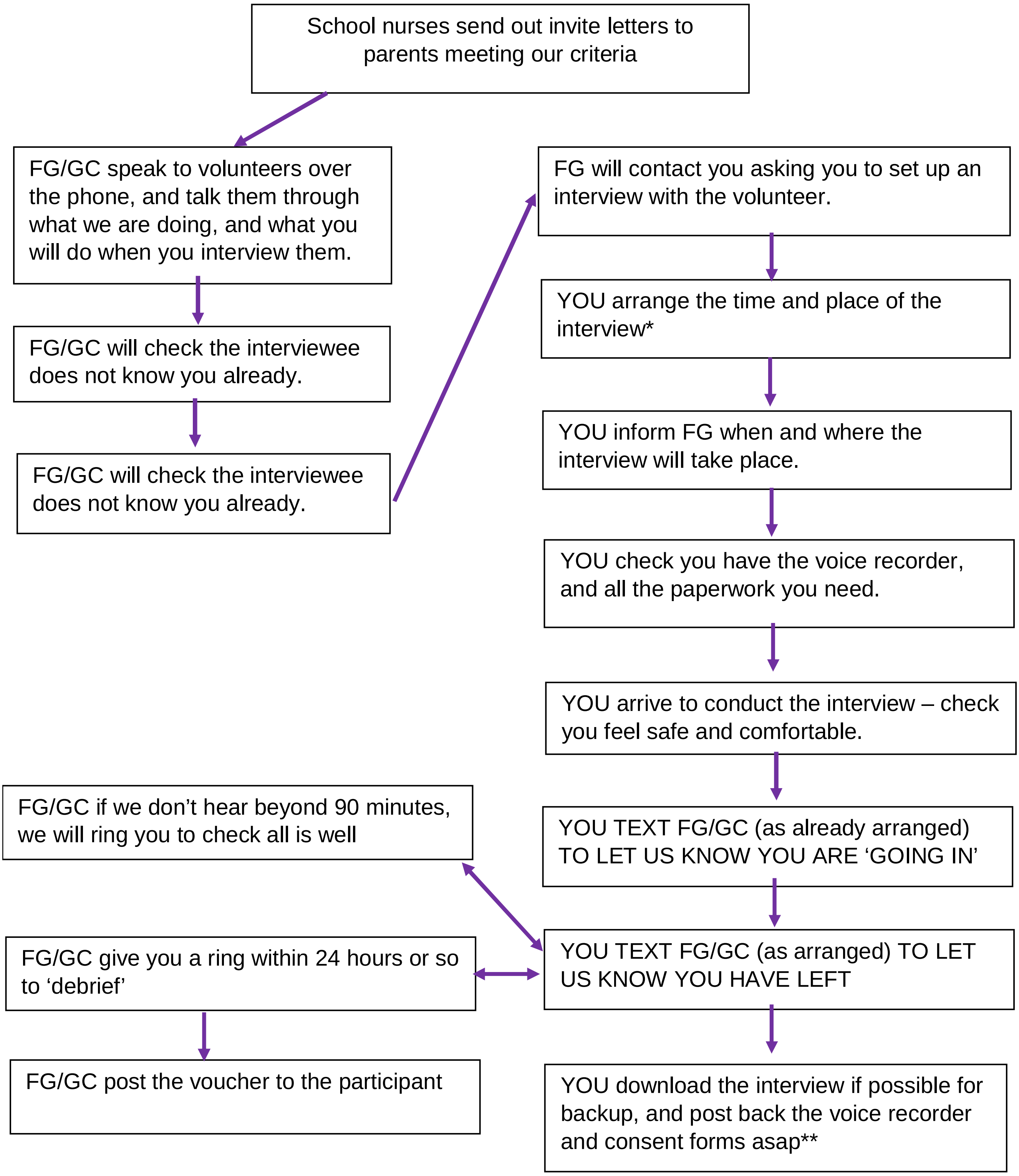

* If you would rather not contact the volunteer yourself, we can do this for you, but would need a clear indication of when you are available etc.

** Posting the voice recorders is a pain, but otherwise we can’t make sure the data is safe. It is likely one of our wider research team may visit Swindon during this time, so we may arrange for her to meet you to download the data instead, if and when this is convenient for you.

Contact: Fiona Gillison: f.b.gillison@bath.ac.uk

Appendix 5: Post-training quiz

- Red text indicates expected answers/responses and will be deleted before circulation x

1. During the interview, the person you are interviewing tells you that they are very worried about their child, and is clearly upset (gets a bit tearful or angry). What do you do?

   Tick yes or no for all that you think apply – there may be more than one right answer

	Yes	No
a) Carry on with the interview questions – don't try to get involved with what is upsetting them		x
b) Try to help/advise them yourself		x
c) Acknowledge that they are upset, but that you are not the best person to help. Given them a 'leave behind sheet' and encourage them to contact relevant service/professional	x	
d) Manage as best you can to calm them down, and talk to us about it later.	x but c) would be better	

2. Jane is a parent who has been trained to do interviews like you. She carries out an interview with Debbie, which she finds very interesting. She learns about lots of new things going on near her, and realises she and Debbie have a friend in common.

	Yes	No
a) Jane is walking with a friend when she bumps into Debbie a few weeks after the interview. They stop to say hello. Is it OK for Jane to tell her friend how she and Debbie met?		x
b) Is it OK for Jane to mention Debbie, and how they met, to their mutual friend?		x
c) Is it OK for Jane to talk about the new activities she has found out about to other friends, as long as she doesn't mention Debbie's name?	x	
d) During the interview, Debbie told Jane about the way she finds works best to avoid arguments with her children at meal times. Is it OK for Jane to talk about this idea to her other friends, as long as she doesn't mention Debbie's name?	x – for discussion, better if not, need to be 100% sure it can't be traced back	

Contact: Fiona Gillison: f.b.gillison@bath.ac.uk

3. If someone doesn't want you to record the interview, what are your options?

	Yes	No
a. Carry on with the interview without making the recording		x
b. Explain that you need to make the recording or you won't be able to accurately remember what is said, without the record we can't use the interview in our study. Explain that it is fine that the person doesn't want to be recorded, but you won't be able to do the interview.	x	
c. Make the recording anyway without the person noticing.		x

Appendix 6: Post training feedback sheet / telephone questions

Parents Interviewing Parents (PIP) project

Feedback from Session X

What is the most interesting/important thing you've taken away from today's session?

Is there anything you are still unsure about?

What would you like more of next time?

Note: We planned to use the sheets above after each session, but in practice more often took verbal feedback after each session. This was in part, as we only had 4-5 participants, so there was little anonymity (which may be the advantage). Written feedback was obtained via email after course end using the form on the following page.

Parents Interviewing Parents (PIP) project

Evaluation Questions – emailed post training

We would like your open and honest feedback so that we can learn from and improve the course for the future. Fiona and Gerry will anonymise your comments for evaluation purposes and they will NOT be shared with other group members.

If you have specific comments on each workshop, that would be helpful, but if not, please go to question 4, 5 and 6 for general feedback.

1.Comments on Workshop 1: Background, intro to research methods, interview skills, listening exercise

---

2. Comments on Workshop 2: Angie's experience, ethics, role of interviewer, skills practice

---

3. Comments on Workshop 3: skills recap, procedures/protocol, practice with interview script

---

4. Were there any aspects of the course that you found ….

(You might want to comment on the role play, or the group dynamics…)

a) difficult or challenging?

---

b) enjoyable or helpful?

---

5. How prepared (or not) do you feel for doing your first interview?

---

6. Any other comments else that might help us improve this training course in the future?

Contact: Fiona Gillison: f.b.gillison@bath.ac.uk

Appendix 7: Cluster framework of themes

Description of clusters of codes from the participant interviews:
Examples retained from the Parents Interviewing Parents study

Cluster	Description
1. Recognition that child is overweight	• How the parent became aware that the child was overweight<br>• Memory (or not) of receiving an NCMP letter<br>• Discussions 'looking back' at beliefs about the child's weight at an earlier time
2. Parents' health beliefs	• Parents' belief as to whether their child is/was overweight<br>• Parents' beliefs about whether or not the child's weight status will change if nothing is done.<br>• Beliefs about the dangers (or not) of being overweight.
3. Parent's role and responsibility	• Parents' views on the legitimacy of measurement /professional involvement in weight loss – should we be weighing children in the first place, and telling parents their children are overweight?<br>• Parents' perceptions of their personal responsibility for making sure their child has a healthy weight.
4. Protection from knowledge	• Not letting a child know as a means of protecting them<br>• Concerns over the risks to child's wellbeing if weight was talked about, or made an issue, within the home.<br>• Parents' views on whether or not their child should be made aware that they are overweight.
5. Protecting childhood	• Discussions about the rights of a child to have a 'normal' childhood, to do what other children do, without being worried about their weight.<br>• May link to cluster 3 - issues about concern for wellbeing if children are made aware that they are overweight / made to feel different
6. Child's role	• The degree to which parents expect the child to be in control of their choices around weight (e.g., what they eat and drink outside the home)<br>• Parents' views of the child's competency to control their eating and drinking.<br>• Degree to which parents feel a child's weight is the child's own responsibility.<br>• The degree to which parents involve the child in making lifestyle changes (e.g., gets them involved in food preparation etc.)
7. Social support	• Recognition of the importance of social support (within the family, or from groups)

	• Peer influences on the child and their weight-related activities (positive or negative)
8. Stigma	• Views of fairness/discrimination against people who are overweight in general.<br>• Discussion of whether weight concern stems from trying to force everyone to be a certain size for aesthetics, rather than for health reasons.
9. Helpfulness of professional support	• Parents' reports of services and whether or not they have been helpful.<br>• Parents' reports of commercial organisations and whether or not they have been helpful.
10. Barriers and enablers	• Suggested tips and tactics.<br>• Changes that the family has made *on purpose* to try and reduce the child's weight<br>• Changes that the family has made that have had an impact on the child's weight, even if that wasn't the purpose<br>• Discussions of how having a child who is overweight has impacted family life.<br>• Barriers to making lasting changes.

Appendix 8: Analysis process in pictures

Step 1: Key points raised by the peer researchers

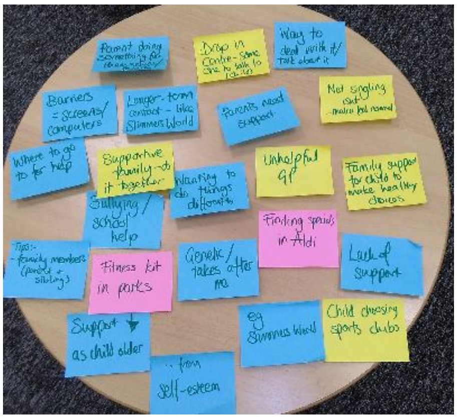

…were combined with…

Step 2: Codes generated by the university research staff

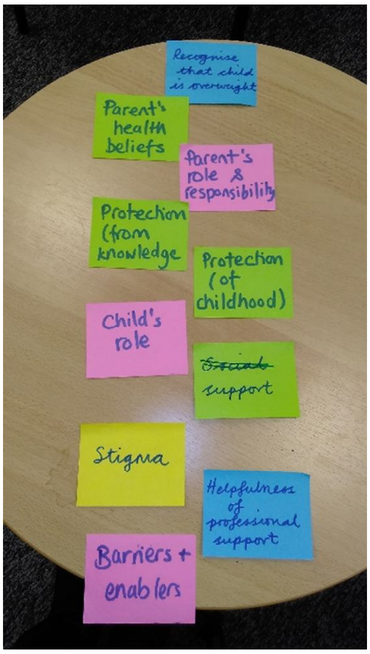

…which led to…

Step 3: Detailed clarification of each cluster of concepts

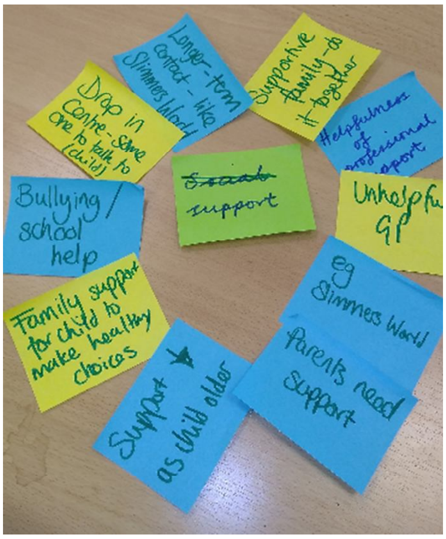

Step 4: Compilation of contributions into three, clearly articulated themes

…resulting in…

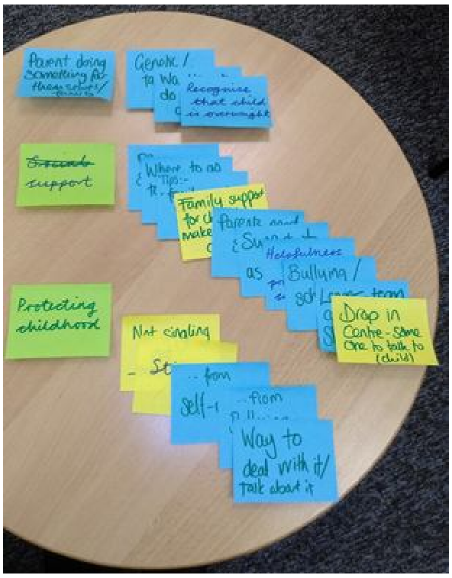

Contact: Fiona Gillison: f.b.gillison@bath.ac.uk
